# Advanced strategies to thwart foreign body response to implantable devices

**DOI:** 10.1002/btm2.10300

**Published:** 2022-03-02

**Authors:** Simone Capuani, Gulsah Malgir, Corrine Ying Xuan Chua, Alessandro Grattoni

**Affiliations:** ^1^ Department of Nanomedicine Houston Methodist Research Institute Houston Texas USA; ^2^ University of Chinese Academy of Science (UCAS) Beijing China; ^3^ Department of Biomedical Engineering University of Houston Houston Texas USA; ^4^ Department of Surgery Houston Methodist Hospital Houston Texas USA; ^5^ Department of Radiation Oncology Houston Methodist Hospital Houston Texas USA

**Keywords:** biomimetic, foreign body response, immune modulation, implantable devices

## Abstract

Mitigating the foreign body response (FBR) to implantable medical devices (IMDs) is critical for successful long‐term clinical deployment. The FBR is an inevitable immunological reaction to IMDs, resulting in inflammation and subsequent fibrotic encapsulation. Excessive fibrosis may impair IMDs function, eventually necessitating retrieval or replacement for continued therapy. Therefore, understanding the implant design parameters and their degree of influence on FBR is pivotal to effective and long lasting IMDs. This review gives an overview of FBR as well as anti‐FBR strategies. Furthermore, we highlight recent advances in biomimetic approaches to resist FBR, focusing on their characteristics and potential biomedical applications.

## 1 INTRODUCTION

**FIGURE 1:**
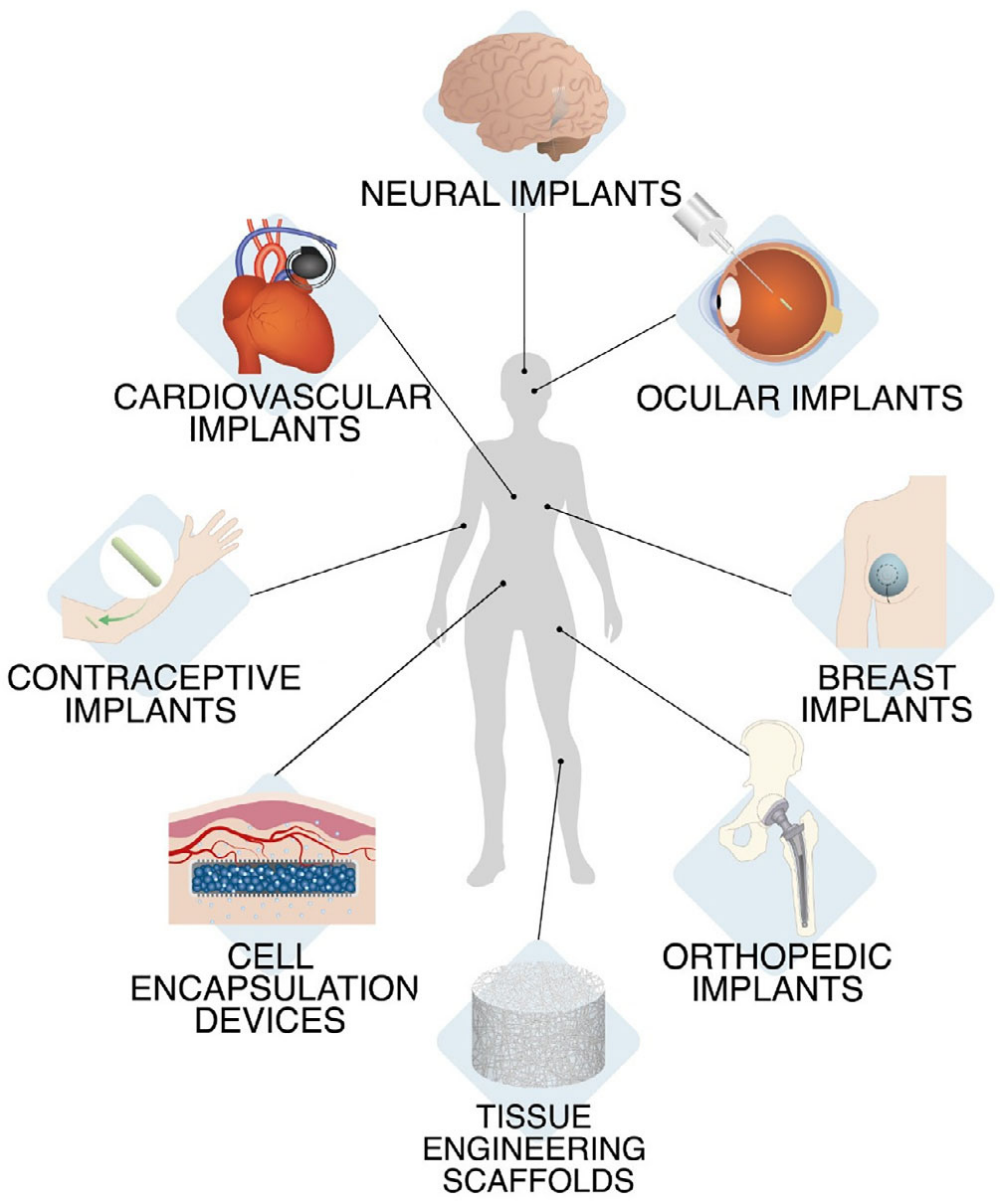
Examples of implantable devices

In the past years, the rising demand for implantable medical devices (IMDs) has been fostered by advances in manufacturing technologies and biomaterial science. This trend is mainly driven by the increasing geriatric population, more prone to chronic conditions, and the increased demand for organ transplantation.^1^, ^2^ Orthopedic prosthesis,^3^, ^4^ breast implants,^5^, ^6^ neural stimulators,^7^, ^8^ cardiovascular devices^9^, ^10^, ^11^ and stents,^12^ ocular and cochlear implants,^13^, ^14^, ^15^ tissue engineering scaffolds,^16^, ^17^ and biosensors^18^, ^19^, ^20^ are only some widely used examples of clinically approved IMDs (Figure 1). Furthermore, IMDs can be utilized as self‐regulated drug delivery and cell encapsulating systems that allow controlled sustained therapeutic delivery and cell engraftment.^21^, ^22^, ^23^, ^24^, ^25^, ^26^ The global IMDs market is expected to grow at a compound annual growth rate of approximately 6.9% from 2020 to 2027 and reach a market value of nearly US$ 155 billion by 2026.^1^ However, despite many advantages that these devices potentially offer to medicine, and increasing demand in the market, most implants fail to meet the implantable devices biocompatibility criteria due to the foreign body response (FBR).

FBR induces the formation of a capsule‐like dense fibrous tissue that isolates the device. FBR consists of a complex series of immune defense mechanisms against foreign material. Upon implantation, the first event is the adsorption of blood plasma proteins, predominantly albumin, and fibrinogen, on the material.^27^ Depending on the material surface properties, proteins undergo conformational changes, resulting in the opening of protein recognition patterns that attract the innate immune system cells; neutrophils, monocytes, and macrophages. Neutrophils are the first‐line responders, as their recruitment to the implantation site happens 2 days postimplantation.^28^

**FIGURE 2:**
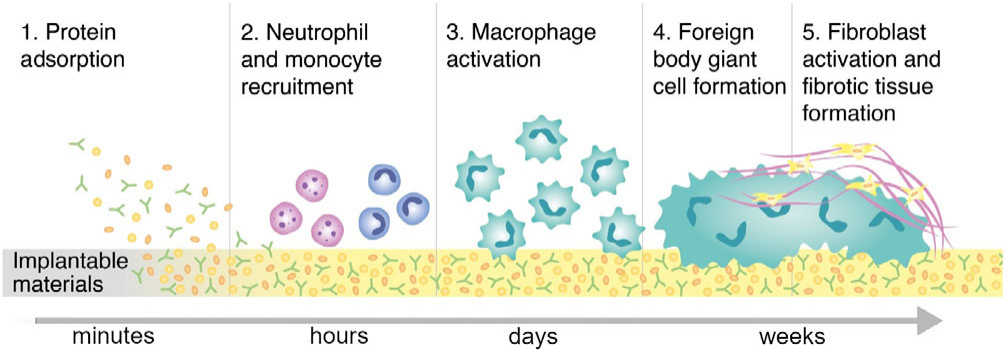
Stages of foreign body reaction and fibrotic tissue formation

This first phase of acute inflammation usually lasts for a week and resolves shortly thereafter. In presence of a foreign body, namely, an IMD, the acute inflammation persists and leads to chronic inflammation.^29^ This phase is marked by monocyte infiltration and macrophage activation, and lasts for approximately 3 weeks.^30^ Macrophages are critical components in capsule formation.^31^ When activated during the inflammation period, these cells are classified as M1 and M2 phenotype macrophages. M1 macrophages secrete proinflammatory cytokines (including interleukin‐1 (IL‐1)) and chemokines,^32^, ^33^ while M2 macrophages upregulate the anti‐inflammatory pathway and tissue remodeling. In the initial stage of immune reaction to tissue injury, the M1 phenotype population is predominant. As the chronic inflammation resolves, macrophage polarization shifts into M2 phenotype and the natural wound healing process. However, in the presence of foreign bodies, such as IMDs, this process is delayed, and proinflammatory macrophage proliferation continues. Macrophages attempt to eliminate the implant via phagocytosis by secreting reactive oxygen species (ROS) and matrix metalloproteinases.^34^ However, in the case of slowly degradable or nondegradable implants, the continuous presence of the device and the inability of macrophages to eliminate the implant promotes the fusion of macrophages into foreign body giant cells (FBGCs).^35^ Antigen presenting proinflammatory macrophages also induce adaptive immune system cells, B lymphocytes, and T lymphocytes to secrete proinflammatory cytokines such as interleukins to induce fibroblast activation. Following the activation of fibroblasts via secreted chemokines and cytokines, weak focal adhesion of the cells on the material surface triggers differentiation of fibroblasts into myofibroblasts via tensile forces. This process is characterized by α‐smooth muscle actin (α‐SMA) expression in intracytoplasmic stress fibers, which implies high contractile activity,^36^ and by secretion of collagen by myofibroblasts. Ultimately, they create a dense, avascular collagen fiber network called fibrotic tissue that encapsulates the device^37^, ^38^ (Figure 2). This tissue blocks the implant–host tissue interaction, which may impair the implant function and subsequently reduce the implant lifetime.^39^ Lack of vascularization, for example, reduces blood supply, limiting oxygen and analyte diffusion, and obstructs drug delivery.^40^ Thus, understanding the tissue response to implantable materials in depth could be leveraged to achieve specific functions (e.g., engraftment) or avoid undesired effects (overt fibrosis) to meet the clinical needs.

**FIGURE 3:**
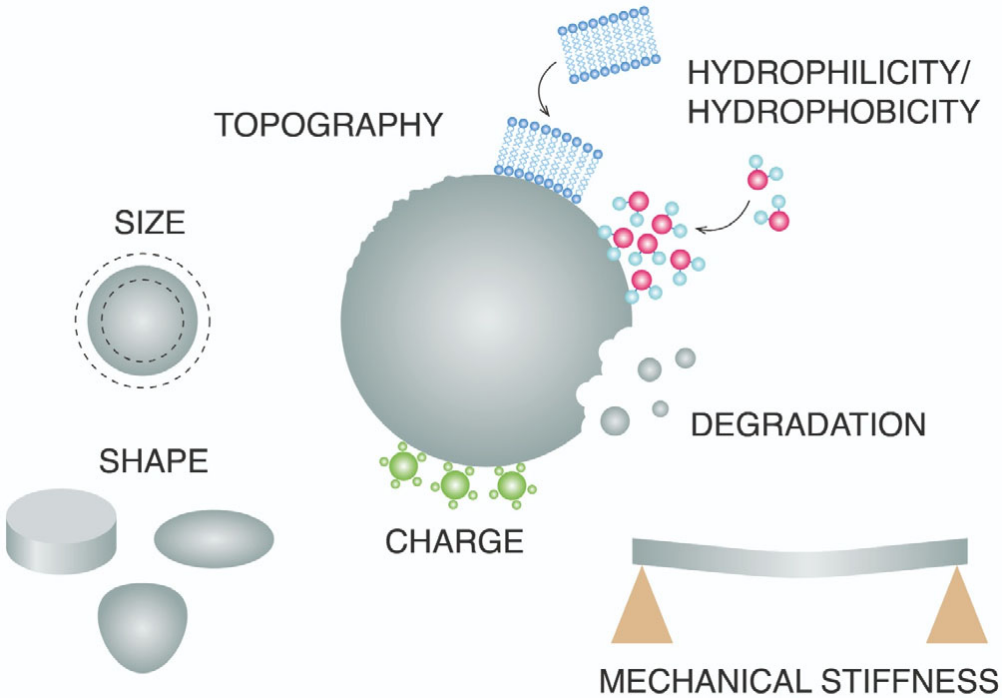
Implant properties that affect the FBR. FBR, foreign body response

FBR and subsequent fibrotic encapsulation contribute to the failure^41^ of many devices, including biosensor,^42^ coronary stents,^43^ breast implants,^44^ encapsulated tissues/cells drug delivery systems,^34^ and ocular implants,^45^ endangering the health of the patients (Table 1). For example, the failure rate of breast implants alone due to the FBR is 30%,^58^ and the failure rate of all other implantable devices is conservatively estimated to be 10%.^66^ Notably, solving this critical clinical challenge could eliminate nearly $10 billion in cost to the healthcare system annually. Therefore, there is a clear need for IMDs design principles that focus on device parameters, such as size, shape, surface topography, mechanical stiffness, and wettability^67^ (Figure 3), critical for the FBR.

**TABLE 1:** Common FBR‐related issues of IMDs

IMD category	FBR‐related issues	References
Cardiovascular implants	Granulomatous reaction to cardiovascular implantable electronic devices (CIED) Fibrosis‐related CIED replacement complications Thrombosis caused by stents or artificial valves	46, 47, 48, 49, 50, 51
Neural implants	Microelectrode arrays (MEAs) recording failures Insertion trauma Giant cell formation around platinum electrodes	52, 53, 54
Ocular implants	Anterior and posterior capsule opacification Inflammation Fibrous proliferation	55, 56, 57
Breast implants	Capsular contracture Granuloma formation Breast implant‐associated anaplastic large‐cell lymphoma	58, 59, 60
Orthopedic implants	Bone resorption Giant cell formation Chronic inflammation	61
Contraceptive implants	Implant extrusion	62
Cell encapsulation devices	Fibrosis and isolation of the implant Cell isolation and hypoxia	63, 64
Tissue engineering scaffolds	Necrosis or inflammation induced by degradation products Inflammation caused by xenogeneic materials	17, 65

Abbreviations: FBR, foreign body response; IMD, implantable medical device.

This review first focuses on the characteristics of implantable devices and how these affect local tissue remodeling in response to immune modulation. Second, repercussions of FBR on implant performances is examined, with emphasis on drug delivery and cell encapsulation devices. Finally, this work discusses recent advances in biomimetic strategies, adopted to mitigate the FBR. More specifically, this review covers solid, nondegradable, implantable macrodevices for long‐term deployment. Cardiovascular devices bear additional complexities related to their blood‐contacting nature, are thoroughly discussed by other groups, and will not be covered herein.^68^, ^69^, ^70^, ^71^, ^72^, ^73^, ^74^, ^75^, ^76^ Similarly, micro‐ or nanoparticles and bone and joint replacements are out of the scope of this review as they have been extensively reviewed elsewhere.^77^, ^78^, ^79^, ^80^, ^81^, ^82^

## 2 IMPLANT PROPERTIES AFFECT THE DEGREE OF HOST RESPONSE

Biomaterial surface properties determine the protein interaction level and biological response of immune cells, particularly the fate of macrophage polarization. Understanding biotic–abiotic interaction is of profound importance in designing implantable biomaterials with immunomodulatory properties. Tailoring the surface characteristics such as roughness, hydrophilicity, charge, size, shape, and mechanical stiffness has a potential impact in changing the direction of FBR towards the tissue repair process. In this section, we will cover the implant material parameters that induce different immune‐mediated FBR.

### 2.1 Surface topography

Surface topography is an essential aspect of medical implants that plays a pivotal role in material‐host tissue integration.^83^ It regulates the density of adsorbed protein on the surface and its interaction with the surface, which induces inflammatory cytokine secretion and macrophage fusion. Altering the surface topography at micro/nano levels can tune the degree of biofouling, focal cell adhesion, proliferation, and ultimately regulate fibrotic capsule formation.^84^, ^85^ For instance, it has been confirmed that osteoblastic cell adhesion, growth, and proliferation are correlated to the surface roughness of Ti.^86^ Different surface roughening methods such as sandblasting or acid etching on Ti, alter the surface topography which can induce proinflammatory cytokine secretion and macrophage activation in vitro.^87^ Polycrystalline diamond coating through chemical vapor deposition on three‐dimensional (3D)‐printed Ti scaffolds can cause higher nanoscale roughness similar to native bone (~66 nm), but similar microscale roughness (25 μm) compared to uncoated Ti scaffolds. Uncoated and coated Ti scaffolds prompted the formation of a fibrotic capsule with similar thickness in rats after 4 weeks, indicating the predominant effect of microscale roughness on FBR.^88^

Different configurations of polytetrafluorourethane (PTFE), a hydrophobic polymeric material widely employed in cardiovascular implants^89^ have an influence on macrophage behavior in vitro.^90^ This material has been studied in flat, expanded, and electrospun arrangements. Electrospun PTFE with a surface roughness of 1.08 μm reduced the macrophage cell attachment and FBGCs formation compared to flat (roughness 0.17 μm) and expanded (roughness 0.37 μm) PTFE.

In another study, poly(2‐hydroxyethyl methacrylate) (pHEMA) hydrogel scaffolds with either nonporous, 34‐, and 160‐μm porous features were implanted subcutaneously in mice for 3 weeks.^40^ pHEMA scaffolds with 34 μm porosity elicited a less dense capsule and increased vascularization.

Silicone is a biocompatible polymer widely implemented as an implant material for many applications, including tissue engineering and drug delivery.^25^ Despite its widespread utilization, silicone does not allow the medical devices to fully integrate with the host tissue and surrounding blood microenvironment. As a result, inflammatory response and fibrosis are inevitable for bare silicone polymer.^91^ Creating micron‐scale hexagonal pits with a diameter range between 3 and 20 μm on silicone surfaces can significantly reduce fibroblast and macrophage adhesion in vitro.^85^ Varying focal cell adhesion and myofibroblast activation on uncoated, entirely collagen‐coated, or micropatterned silicone implants can induce different fibrotic tissue responses.^92^ For instance, unlike counterparts, micropatterned structures with 4 μm × 2 μm features showed a lower surface tension transferred to cells and successfully kept the fibroblasts at a noncontractile state. In addition, in microarrays with equal spacing (5 μm), the density of fibroblasts attached to surfaces exhibiting 4 μm × 2 μm features was higher (266 ± 21 cells/mm^2^) compared to the ones with 10 μm × 2 μm (196 ± 5 cells/mm^2^) and 20 μm × 2 μm (210 ± 22 cells/mm^2^) features. Moreover, in vivo rat studies revealed that fibrotic capsule thickness was lower in micropatterned (37 ± 11 μm) surfaces compared to uncoated (50 ± 19 μm) and fully coated implants (90 ± 37 μm).

Doloff et al. examined the FBR of clinical polydimethylsiloxane (PDMS) breast implants with various surface roughness (0–90 μm) in mice, rabbits, and in human samples obtained from revision surgeries.^93^ Gene expression levels of proinflammatory markers indicate that SmoothSilk implants with a roughness of 4 μm exhibited a reduced fibroblast spreading and macrophage population compared to the smoother (roughness <1 μm) and rougher (roughness 15, 30, and 90 μm) counterparts while regulatory T cell activation, a key factor to induce prohealing stage. Furthermore, implants with 4 and 15 μm of surface roughness induced thinner fibrotic capsule formation. In another study, PDMS implants with acellular dermal matrix (ADM) topography (484 nm roughness) were compared to commercially available smooth and highly rough (8.24 μm) macrotextured PDMS surfaces.^94^ Implants with ADM topography prompted an enhanced focal adhesion and spreading morphology of breast‐derived fibroblasts in vitro. In addition, expression of proinflammatory cytokines IL‐8, tumor necrosis factor‐α (TNF‐α), transforming growth factor‐β1 (TGF‐β1), and HSP60, was downregulated, indicating that reproducing the extracellular matrix (ECM) topographical cues can be a promising approach in reducing FBR.

Polycaprolactone (PCL) is a widely applied material due to its biocompatibility, slow degradation kinetics as well as cost‐effectiveness, and easy modification in biomedical applications, particularly tissue‐engineered scaffolds.^95^ Phase evaporation, used to increase the roughness of PCL films (~1 μm), can create a higher surface area and hydrophobicity, which allows higher bovine serum albumin (BSA) adsorption on the surface.^96^ In addition, rougher and more hydrophobic PCL surfaces present more anchoring points for mouse calvaria‐derived preosteoblastic cells (MC3T3‐E1) in vitro. Furthermore, macrophage fate can be determined through the alteration of the surface topography. PCL films microgrooved under near‐infrared irradiation can trigger macrophage elongation, resulting in increased M2 polarization compared to the flat PCL surface both in vitro and after implantation in a rat model.^97^ Through the electrospinning process, PCL can be produced in aligned nanofibers with a high aspect ratio (length/width). For example, electrospun PCL/collagen nanofibers resembling ECM texture can promote the cellular fate toward the healing stage.^98^

Nanofibers arrangement in electrospun scaffolds influences the microscale surface topography. In comparison with the randomly aligned nanofibers, poly(lactide‐co‐glycolide)–poly(3‐hexylthiophene) (PLGA‐PHT) blend electrospun scaffolds with axially aligned structure demonstrated mild inflammatory response in rats, as well as slow degradation kinetics.^99^ In a comparative study, PCL, poly(lactic acid) (PLA), and PDMS parallel nanoimprinted parallel gratings with line width ranging from 250 nm to 2 μm were cultured with RAW 264.7 macrophages and then implanted in rats for 21 days.^100^ Macrophages were responsive only to features in the microscale and independent of the material. Surfaces with 1 μm gratings reduced TNF‐α and vascular endothelial growth factor (VEGF) levels compared to submicrometric and planar gratings, hinting to a decreased proinflammatory polarization. In vivo, larger gratings elicited a reduced macrophage density and cellular fusion, and thinner fibrotic capsule consistently throughout the materials.

Patterning density can also influence focal cell adhesions. A dense microstructure on hydroxyapatite ceramic artificial lamina showed a reduced fibrotic tissue formation 6‐week postimplantation in rabbit vertebra compared to a lesser dense structure.^101^ Similarly, biocompatible nanotextured tantalum (Ta)‐modified silicone implants significantly reduced fibrotic capsule thickness.^102^ In contrast, bare silicone implants generated a denser collagen network and thicker fibrotic pattern after 8 weeks of subcutaneous implantation in a mouse model.

Recently, Vassey et al. developed a high throughput screening technology platform, named TopoChip, that applied an algorithmic approach and machine learning principles to investigate the effect of 2176 distinctive micropatterned surfaces on the phenotypic changes of human monocyte derived macrophages.^103^ The study found that micropillar sizes ranging from 5–10 μm in diameter can enhance macrophage adhesion and a combination of micropillar size and density can modulate their phenotype.

Collectively, these findings suggest that surface roughness or modified surface topography obtained by adjusting height and depth of surface features can influence the FBR formation through modulation of cell adhesion patterns. Roughness smaller than 1 μm appear to have little to no effect on FBR mitigation, while surface features in the range of 1–4 μm show a potential to ameliorate implant integration. Spatially confined surfaces with a diameter smaller than the size of an immune cell can limit the spreading and activation of proinflammatory cells on the material surface.^85^ Although tuning surface topography could reduce FBR, it is of paramount importance that the overall mechanical and functional properties of the device remain unaltered.

### 2.2 Surface charge

The surface charge can influence the protein adsorption and the interactions between immune cells and the material at different stages of FBR. In particular, adsorption is dictated by the overall charge, present on the surface of the material rather than by atomic‐scale electrostatic interaction.^104^, ^105^, ^106^ Moreover, the balance between the surface isoelectric point and the pH of the surrounding fluid defines the charge at the material and fluid interface. Thus, a pH below or above the surface isoelectric point generates a positively or negatively charged surface, respectively.^107^ Furthermore, electrostatic interactions between negatively charged cells and charged material surfaces contribute to FBR to biomaterials.

Hunt et al. were among the first, in 1996, to study how the material surface charge affects the inflammatory response. The group reported that poly(ether)urethane with a negative net surface charge could reduce neutrophil infiltration and influence macrophage activation.^108^ On the other hand, a positive surface charge appears to boost the inflammatory response towards implanted biomaterials.^109^ Notably, positively charged alginate/poly(ethylene imine) hydrogel showed higher cell adhesion and thicker fibrotic capsule than negatively charged hydrogels. In another study, the FBRs to hydrogels bearing opposite charges were also compared.^110^ Positively charged hydrogels elicited an acute inflammatory response, characterized by higher infiltration of immune cells, collagen deposition, and neovascularization. Conversely, negatively charged hydrogel caused minimal inflammation, resulting in the absence of collagen and neovascularization. In an in vitro study, Lee et al. tested the effect of nanostructured titanium surfaces modified with divalent cations on macrophage attachment and proliferation in vitro.^111^ The combination of surface treatments significantly reduced cell adherence after 24 h of incubation compared to the unmodified nanostructured surface. In addition, increased macrophage polarization towards the wound‐healing M2 phenotype was observed on the ion‐modified surfaces.

One of the molecules most utilized to obtain biocompatible biomaterials is polyethylene glycol (PEG) which provides a shielding effect. PEG is negatively charged, and it is known to protect biomaterials against nonspecific protein adsorption.^112^ PEG was used to coat a polymer using a layer by layer (LBL) technique to create a material for on‐demand dexamethasone (DEX) release.^113^ Polymer coating consists of cationic polyelectrolyte [poly(diallyl dimethylammonium chloride); PDDA] and anionic polyelectrolyte (polystyrene sulfonate; PSS) and PEG grafted on DEX via ester bonds. LBL coating was tested on PCL scaffolds. The authors also investigated the fibrosis attenuation property of the polymer coating on the skin wound‐healing model. Fibrosis‐related α‐SMA expression from myofibroblast was significantly reduced with the PEG‐DEX modified polymer coating than the unmodified PSS/PDDA. Although it is widely used as an antifouling approach due to its stealth character, some studies demonstrated that PEG is insufficient to prevent fibrosis^114^ because of its poor long‐term stability at the material–tissue interface. First, PEG‐modified surfaces are prone to oxidation in the physiological environment,^115^ and this eventually causes the degradation of the PEG chains. Second, ROS generated by immune cells create peroxide linkage that causes the decomposition of PEG and reduction in the PEG chain density. Therefore, alternative polymeric approaches with long‐term stability profiles have emerged.^116^

A neutral net charge is thought to prevent protein adsorption to material surface.^117^ Zwitterionic materials are made of moieties bearing positive and negative charges presenting specific structure results in a balanced neutral charge. This creates a hydration layer via electrostatic interactions with water molecules, thus, exhibiting antifouling properties by effectively impeding protein adsorption on the material surface.^118^, ^119^ Recently, zwitterionic materials, such as phosphorylcholine, carboxybetaine, sulfobetaine, have gained much attention due to their ultralow fouling feature.^120^, ^121^ Accumulating body of evidence suggests that modification of implantable material surfaces with such coatings is significantly effective at mitigating fibrosis and increasing macrophage polarization.^122^, ^123^ For instance, Zhang et al. reported that, unlike pHEMA hydrogels, carboxybetaine zwitterionic hydrogels can reduce the capsule formation for 3 months when implanted in mice.

Furthermore, zwitterions induce anti‐inflammatory M2 macrophage expression.^124^ In a recent study, neural microelectrodes were coated with zwitterionic layer consisting of poly(sulfobetaine methacrylate).^125^ The treatment prevented protein adsorption, fibroblast, and microglia attachment on the electrodes and remained stable in vitro for 4 weeks. Furthermore, in a short‐term implantation test, the coated microelectrodes significantly reduced microglial surface coverage compared to uncoated controls. In addition, zwitterionic‐mimicking materials can be developed by assembling oppositely charged macromolecules, such as the balanced charged alginate/poly ethylenimine hydrogel.^109^ After 3 months subcutaneously implanted in mice, the hydrogel showed significant antifouling properties, diminished the FBR, and subsequent capsule formation.

It can be challenging to isolate and study the surface charge as a single factor avoiding other properties, such as surface wettability.^126^ Nevertheless, negatively charged surfaces appear to elicit a milder response, followed by thinner capsule deposition and limited neovascularization when compared to positively charged counterparts. Furthermore, surfaces exhibiting a neutral charge prevent protein adsorption and significantly reduce the FBR.

### 2.3 Surface wettability

Protein adsorption, the first stage of FBR, is generally energetically favorable towards hydrophobic surfaces.^127^ On the contrary, removing the water molecules from the hydrophilic surfaces bears a higher energy barrier^114^ demonstrating protein repellent features.^128^ In this aspect, materials with hydrophilic surfaces can govern protein adsorption as well as immune response modulation. For instance, modifying the cationic active site of PDMS surfaces with negatively charged hydrophilic polysaccharides, such as hyaluronic acid or sulfated fucoidan^129^ lead to reduced BSA and fibrinogen adsorption in vitro on the material surface due to the low electrostatic interactions and high hydration forces. Studies have also demonstrated that carboxylic acid (–COOH), hydroxyl (–OH), and amine (–NH_2_) functional groups can enhance hydrophilicity.^130^ Surface hydrophilicity can be improved by applying surface modification methods such as ultraviolet, plasma treatment, or ion beam implantation. In a recent report, O_2_ plasma‐assisted and chemical conjugation of PDMS biomedical implants with –COOH bearing itaconic acid and –NH_2_ bearing gelatin considerably enhanced the surface hydrophilicity. The treatments led to a significant reduction in capsular thickness and collagen density in rats model for up to 8 weeks compared to bare surfaces.^131^

Contrarily, self‐assembled monolayer biomaterial surface with hydrophobic methyl (–CO_3_) groups significantly provoked the inflammatory response in macrophage/fibroblast in vitro coculture system compared to the hydrophilic/anionic –COOH model surfaces.^132^ Modifying the surfaces with hydrophilic functional groups can induce macrophage polarization towards the M2 phenotype. Grafting poly‐d‐lysine with a hydrophilic –NH_2_ group onto the unsaturated polyurethane (PU) films can activate the downstream signaling pathways of M2 signature.^133^ Similarly, after implanting cylindrical Ti implants with rough, smooth, rough‐hydrophobic, and rough‐hydrophilic properties in mice femoral canal, rough‐hydrophilic implants demonstrated higher levels of macrophage induced T helper 2 cell population, an indicator for the prohealing stage after 3 days.^134^ By Day 7, the same implants showed a higher macrophage population with enhanced mesenchymal stem cells (MSCs) recruitment due to the secreted cytokines from M2 phenotype.

In a 2007 study, fibrinogen, BSA and human coagulation factor XII with adhesion forces on plasma‐treated low‐density polyethylene substrates exhibited a step decrease on hydrophilic surfaces presenting a water contact angle lower than 60°–65°.^135^ Based on these findings, the authors speculated the existence of a threshold to identify protein‐adherent and nonadherent materials.

PCL, a synthetic polymer, has been broadly used to develop vascular grafts and tissue engineering scaffolds. However, due to its intrinsic hydrophobicity, it can cause nonspecific protein adsorption^136^, ^137^ as well as fibrosis. Moreover, PCL hydrophobicity can be further enhanced by increasing its roughness, facilitating BSA adsorption and improving cell adhesion and proliferation in vitro.^96^ Functionalizing the PCL nanofiber surfaces with heparin disaccharide, a hydrophilic GAG, silk incorporated PCL scaffold created via LBL approach, demonstrated lower macrophage recruitment compared to the unmodified PCL or silk functionalized PCL fibers 28 days postimplantation in SD rats.^138^ This indicates that heparin hydrophilicity can reduce the nonspecific protein interaction during the early stages of inflammation. For instance, a correlational study was conducted using model surfaces with different functional groups. Hydrophobic surfaces incubated with serum proteins have been shown to induce macrophage polarization towards the proinflammatory pathway, while hydrophilic surfaces induced an anti‐inflammatory response.^139^ Similar findings are reported for PLA nanofibrous scaffolds functionalized with poly(glycerol sebacate) elastomer for enhanced hydrophilicity. After 28 days of grafting on mice hearts, the scaffold induced neovascularization and elicited a lower inflammatory response than nonfunctionalized PLA scaffold.^140^ Beyond functionalization, fiber arrangement and porosity can affect the wettability of fibrous materials. Polyethylene terephthalate textile implants with porous spun show high hydrophilicity and decreased cell proliferation in vitro compared to less porous counterparts.^141^Overall, these studies underscore the importance of surface wettability on material biocompatibility. Hydrophilic surfaces with mechanically and chemically stable functional groups can be an efficient design approach to govern protein adsorption and the acceleration of the tissue repair process by directing macrophage polarization.

### 2.4 Implant size and shape

Implantable device size and shape characteristics significantly impact the FBR. Polymer microfibers with diameters below a threshold value of 5.9 μm prevent fibrotic capsule formation,^142^ in analogy to micropatterned surfaces discussed in previous sections. The authors speculate the ECM distortion caused by the microfibers might be minute, avoiding cell migration into the void space created by the device. Similarly, another group observed no fibrous capsule and a reduced macrophage population surrounding small fibers (diameter <6 μm).^143^ However, the separation of adjacent collagen layers caused by device implantation creates low‐pressure areas that are subsequently filled by fibrous tissue.^144^ Intuitively, lesser separation will reduce fibrous tissue extent. In a comparative study, the FBR to square shaped multipolymeric membranes (polycarbonate‐based with PU, silicone, and PEG) with different thickness was evaluated. After 7 weeks of subcutaneous implantation in rats, thin membranes (0.3 mm) lead to thinner fibrotic capsule formation than thicker counterparts (2 mm).^145^ Nevertheless, membrane thickness can affect their rigidity, which can ultimately influence the FBR, as it will be examined in the following section.

Conversely, partially contrasting findings are reported by a study from Veiseh et al.^146^ The group evaluated the FBR to a broad spectrum of materials (plastics, metals, ceramic, and hydrogels) with various sizes and shapes. Rodents and nonhuman primates (NHP) were implanted intraperitoneally and subcutaneously with spheres with diameters ranging from 0.3 to 2 mm for 2 weeks. Surprisingly, a significantly reduced FBR was elicited by larger spheres (1.5–2 mm diameters) compared with smaller analogs across all materials, species, and implantation sites. The authors suspect this effect can be induced by the higher curvature on the surface of smaller spheres. Analogous observations resulted from the intraperitoneal implantation of cylindrical alginate implants (1 and 6 mm diameters) in mice.^147^ The smallest devices had increased cellular deposition that caused a thicker fibrotic layer on their surface. Similarly, hydrogel fiber diameter appears to have an effect on cellular deposition. Fiber diameters ranging from 1 to 6 mm showed significantly decreased cellular deposition compared to smaller counterparts.^148^ Nonetheless, discoidal silicone implants with different sizes exhibited comparable inflammatory response and fibrotic capsule thickness.^149^ Thus, most studies concur that implant size affects FBR even though contrasting results were observed. This could be due to different experimental setups, materials, and surface properties.

Avoiding sharp angles and discontinuities is of utmost importance regarding device shape. Matanga et al. studied the tissue response caused by polymeric rods with circular, triangular, and pentagonal cross‐sections.^150^ Acute angles triggered a denser fibrotic reaction due to higher interfacial stress, potentially leading to severe tissue injury. Inversely, circular rods elicited a reduced FBR. These findings are corroborated by Veiseh et al., where spherical implants exhibited a mitigated reaction in contrast to discoidal devices.^146^ Moreover, different fibrotic responses are observed along with the angles of the devices compared to flat areas.^144^ A higher collagen density is present in the capsule near the edges. In contrast, flat regions elicit less stress on the adjacent tissue and, therefore, a milder FBR. Therefore, sharp angles are significant contributors to FBR, and the orientation of the implant can impact fibrotic capsule formation.

Interestingly, a thicker fibrotic capsule was measured around the surfaces parallel to the skin than along the sides with discoidal hydroxyapatite subcutaneous implants, in contrast with the studies presented above.^151^ However, no other relevant literature is available in support of these findings. As the author speculates, the thicker fibrotic capsule adjacent to the top and bottom of the hydroxyapatite disc might be caused by the increased surface area and by chemical stimulation provided by the hydroxyapatite itself.

Overall, these results confirm that the best practice in implant design is refraining from sharp discontinuities and acute angles. Furthermore, implant height and shape curvature are essential contributors to FBR that should be considered regarding implant size. For instance, thinner implants and small spherical or cylindrical implants cause milder and stronger FBR, respectively. Smaller objects have high curvatures, causing thicker fibrotic capsules due to increased inflammation and cellular deposition. However, on the microscale, implant height or fiber diameter <6 μm appears to prevent fibrotic capsule formation. For this reason, the ideal size and shape should be determined depending on the required volume an implant needs to function.

### 2.5 Implant stiffness

One of the parameters of the implant that influences the FBR is stiffness. This mechanical property is a characteristic of the material and it can be measured with the elastic modulus or Young's modulus. It appears that implant materials with analogous Young's modulus to the one of the surrounding tissue can help avoid severe immune response.^152^ Mismatch of Young's modulus at the biotic–abiotic interface is one of the fundamental driving forces in scar tissue formation.^153^ Shear stress due to the stiffness of the material and micromotion in the brain, for example, damage the surrounding tissue resulting in enhanced proinflammatory cell activation, including reactive astrocytes.^154^ Currently deployed brain implants, predominantly silicon implants, are much stiffer^155^ than the brain tissue (~1–30 kPa) and can generate acute FBR that may impact their funciton. Thus, efforts have been devoted to developing softer implants to reduce FBR.

Ecoflex, a silicone‐based material with a low stiffness (20 kPa) was assessed as a mechanically matched brain implant (MMBI) in rats.^156^ MMBIs consistently elicited a reduced level of activated microglia, reactive astrocytes, and neuronal loss than the stiffer PDMS (~1.6 MPa) and silicon (~180 GPa) implants at both 3‐ and 9‐week postimplantation. Ecoflex promoted higher neuronal density and reduced the FBR in proximity of the tissue–implant interface compared to silicon implants. However, there were no significant differences between Ecoflex and PDMS implants. In a similar work, the FBR to silicon and polymeric microelectrodes with different stiffness was evaluated in mice.^157^ Silicon (~150 GPa), polymide (1.5 GPa) and two types of off‐stoichiometry thiol‐enes‐epoxy (OSTE+) probes, OSTE+_Hard_ (300 MPa) and OSTE+_Soft_ (6 MPa) were implanted for 4 and 8 weeks. The stiffest material induced a more severe inflammatory response than the polymer probes, increasing microglial cell and macrophage activation. However, no significant difference was found among polyimide and OSTE+ probes, perhaps indicating that below a certain stiffness, softening the material has minimal impact on inhibiting FBR.

Differently, hard and soft hydrogels, fabricated from 4% and 1% pectin aqueous solution respectively and implanted subcutaneously in rats showed dissimilarities in the induced acute FBR.^158^ Soft hydrogels (14 kPa) elicited lower leukocytes infiltration and circulating levels of proinflammatory cytokines compared to the hard ones (106 kPa). Although there is a mismatch in stiffness between the hard hydrogel and the surrounding tissue, these results are in contrast with the previously discussed findings. However, the authors hypothesized that the difference in acute inflammatory response can be caused by the higher concentration of pectin in the hard hydrogel coupled with its slower degradation kinetics. In another study, PEG‐phosphorylcholine hydrogels with a stiffness ranging from 3 to 165 kPa were implanted subcutaneously in mice. In this case, there was a direct relationship between the stiffness of the hydrogel and macrophages adhesion and fibrotic capsule thickness. Modulation of hydrogels stiffness has also been explored as an approach to develop mechanically matching electronic nerve interfaces for tissue regeneration. Schwann cell proliferation was compared among magnetically templated glycidyl methacrylate hyaluronic acid hydrogels with different stiffnesses.^159^ The hydrogels with mechanical properties similar to fresh nerve tissue promoted cell migration and infiltration within the scaffolds.

Myofibroblast activation, and subsequent collagen deposition, can be suppressed by reducing the mechanical stress generated by implants stiffer than the surrounding tissue.^160^ Coating stiff implants with a layer of soft material that matches the elastic modulus of the host tissue can prompt decreased inflammation and fibrosis in comparison to uncoated implants (preprint).^161^ A stiff silicone rubber core with Young's modulus ~600 kPa was coated with 0.6 kPa polyacrylamide (PAA), 6 kPa PDMS, or 60 kPa PAA. Coated and uncoated implants were implanted for 3 months in subcutaneous tissue and nerve conduits in rats. In both sites, the coated implants showed reduced α‐SMA and CD68 expression in the surrounding tissue than uncoated ones. In addition, a significant decrease in fibrotic capsule thickness was observed in soft‐coated implants. In a similar study, the FBR to soft silicone coating (~2 kPa) applied on stiff silicone implants (2 MPa) was evaluated post‐3 months subcutaneous implantation in mice.^162^ The coated implants elicited a reduced myofibroblast activation and subsequent collagen deposition than the uncoated counterparts. Moreover, the soft‐coated implants showed a reduced TGF‐β1 activation, a profibrotic growth factor that induces myofbroblast contractile activity and leads to the formation of fibrotic tissue.^163^, ^164^

Cell behavior is driven by mechanical stimuli, among other cues. Therefore, it appears that materials with similar stiffness to the surrounding tissue at the abiotic–biotic interface have higher chance to ameliorate the FBR. Modifying implant outer layer can represent a viable strategy to preserve functionality while matching interface mechanical properties. However, this may not be sufficient. As an example, micromotion in neural implants can cause injuries and inflammation, suggesting the need for implants fully matching mechanical properties of surrounding tissues.

### 2.6 Implantation site

Medical device implantation sites can be selected based on implant size and specific function. In addition, differences in FBR related to the implant microenvironment need to be considered. For instance, latex microcapsules implanted intraperitoneally and in the renal subcapsular space induced a different degree of fibrosis.^165^ In the peritoneal space, microcapsules had higher fibrosis deposition, possibly due to the elevated presence of macrophages. Furthermore, severe fibrosis surrounded microencapsulated cells implanted intraperitoneally; none was observed on the counterparts implanted in the subcutaneous space and under the kidney capsule.^166^ In addition, a significant neutrophil population increase was observed in the peritoneal space following microcapsules implantation.^167^ Conversely, PLA and PLA/PCL blend implants elicited similar FBR following subcutaneous and intraperitoneal implantation in rats for 2, 8, and 24 weeks.^168^ In another study, cylindrical PEG hydrogels were implanted in the subcutaneous space, abdominal cavity, and adipose tissue.^115^ The mildest inflammatory response was induced by subcutaneous implants, followed by implants in the abdominal cavity. These abdominal implants showed an increase in macrophage infiltration and few neutrophils. However, the most robust response was observed in the adipose tissue, which is known to be a more hostile microenvironment.^169^, ^170^ While, the intraperitoneal space is an attractive site for cell transplantation due to its abundance of blood vessels, and consequently oxygen, excessive fibrosis can hamper its facile diffusion. Nonetheless, fast tissue integration on implants, a specific trait of this implantation site, might be the desired effect in specific applications.^171^

In a recent study, fibrosis‐generating biomaterials were implanted across different species and sites in an effort to explore the variations in FBR between the subcutaneous space and the immune‐privileged intrauterine environment.^172^ Minimal intrauterine fibrosis was observed in NHP, whereas a strong fibrotic FBR was provoked by the same biomaterials implanted subcutaneously in mice. In this setting, subcutaneous sham surgeries led to negligible fibrosis, excluding tissue disruption as the major factor for FBR discrepancy. Different from subcutaneous implantation, intrauterine placement causes negligible tissue disruption, which could justify FBR discrepancy. Thus, the authors speculate that uterine immune privilege could play a role in minimizing fibrosis. In another study, FBR to collagen discs implanted in the left ventricular epicardium and the subcutaneous space was investigated.^173^ Notably, discs in the epicardium exhibited a stronger inflammatory response with a higher influx of macrophages, PMNs, and angiogenesis. Moreover, distinct subcutaneous locations contributed to differences in fibrotic capsule thickness.^174^ The fibrotic capsule was five times thicker in devices implanted in the middorsal space compared to the scapular site. This discrepancy could be attributed to the different shear forces on the implant that occur in the specific sites; hinting that the microenvironment is not the sole key determinant in FBR variation.^144^ The implantation site is also highly critical for implantable sensors as the FBR can potentially impair the sensor function. For example, intravascular sensors can provide accurate measurements, but activation of the coagulation cascade is a major concern.^175^

Finally, recent studies suggest that the key to understanding the FBR variation at different implantation sites may be to determine the tissue‐resident macrophages population. The behavior of these macrophages is influenced by the niche in which they reside.^176^, ^177^ However, at the moment the role of tissue‐resident macrophages phenotypes in the FBR to biomaterials is not fully understood.^178^

To summarize, the implantation site is strictly dependent on implant function and the desired extent of integration and encapsulation. In addition, the immune microenvironment and shear stress can significantly alter the FBR. For instance, a drug delivery device can benefit from the milder FBR elicited in the subcutaneous space. A thinner and less dense fibrotic capsule will allow for a facile drug diffusion outside of the device. On the other hand, some cell encapsulation devices, which rely on graft revascularization to support its viability, might need a stronger FBR that induces angiogenesis. In this case, the intraperitoneal space can be designated as the ideal site.

## 3 HOST RESPONSE AFFECTS THE IMPLANT PERFORMANCE

### 3.1 The effect of fibrosis on drug and analyte diffusion

**FIGURE 4:**
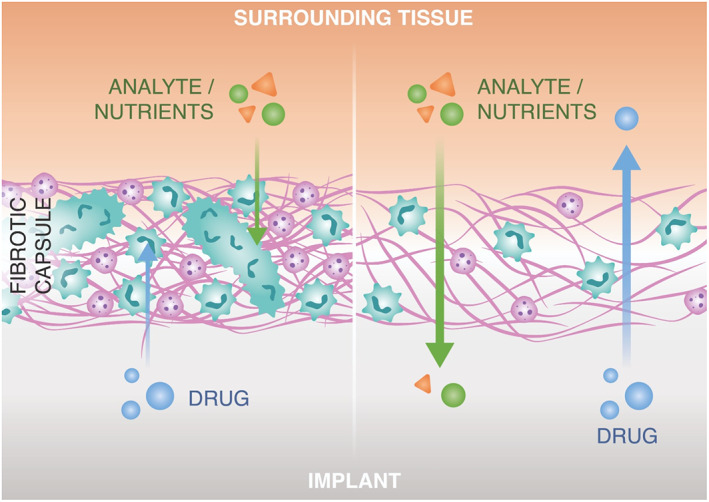
The effect of fibrosis on drug and analyte diffusion

Implantable long‐acting (LA) drug delivery devices are platforms that enable sustained and controlled drug release, and have proved to significantly promote patient adherence.^179^, ^180^, ^181^ Several implantable systems such as degradable, nonbiodegradable polymeric implants and LA in situ forming depots have been tested in vitro and in vivo. While several LA drug delivery device can successfully achieve controlled/sustained drug release, FBR can impair long‐term device performance. Depending on the implantable material chosen, the entity of the fibrosis that surrounds the implant can vary. Moreover, different biomaterials can affect the extent and functionality of FBR‐driven neovascularization.^182^ These factors may affect the drug transport from the implant^183^(Figure 4). For instance, a dense fibrotic network around a LA injectable depot reduced the dissolution and absorption rate of paliperidone palmitate in rats, affecting plasma concentration.^184^ After implantation of PLGA millirods in rat liver, significant deviation in doxorubicin intratumoral delivery was observed due to the fibrotic tissue around the device, 8 days after radiofrequency ablation. Accumulating most of the drug at the ablated region, fibrosis limited the drug transport to the nonablated region.^185^

Drug‐device combination can also exacerbate the immune response. For instance, a 28 day sustained infusion of tenofovir alafenamide (TAF) hemifumarate through a subcutaneous catheter connected to a pump, caused an intense immune response in rats and dogs.^186^ Severe necrosis and infiltration of proinflammatory cells, limited the prolonged drug delivery at therapeutic dosage due to safety concerns. Similarly, Su et al. evaluated the effect of prolonged subcutaneous delivery of TAF through a semipermeable PU reservoir that controls the drug diffusion rate in rabbits and NHP.^187^ After 12 weeks of implantation, due to the focal toxicity, TAF implants induced severe inflammation and fibrosis at the implantation site. These results indicate that in addition to device parameters, drug formulation can also cause FBR, and therefore hamper tissue integrity and sustained therapeutic release from such implants.

Our group developed an array of drug delivery implants based on a nanofluidic silicon membrane that controls therapeutic release. The membrane is coated with silicon carbide to provide long‐term bioinertness.^188^ Unlike aforementioned polymeric implants, local release of TAF from the nanofluidic implants elicited only a slight to moderate reaction around the device. In this case, the FBR did not significantly compromise the drug preventive or therapeutic efficacy in a simian HIV NHP model.^24^, ^189^ In addition, two iterations of the nanofluidic drug delivery implant, made of polyether ether ketone (PEEK) and 6AI4V titanium prompted distinct immune responses in NHP. The devices released 2‐hydroxypropyl‐β‐cyclo‐dextrin‐enhanced cabotegravir (βCAB) in a sustained fashion, and despite a thicker fibrotic capsule surrounded the PEEK implants, the observed βCAB plasma levels were comparable.^179^ Similar results were obtained with long‐term administration of testosterone in a castrated rat model. Implants showed consistent subcutaneous delivery maintaining steady testosterone plasma level over 6 months.^190^ This indicated that the fibrotic capsule developed around the implant did not cause detectable changes in drug release.

FBR can also compromise clinically approved continuous glucose monitoring (CGM) device lifetime. In a typical CGM device, the formation of a dense fibrotic tissue and infiltration of inflammatory cells significantly limits the analyte diffusion (Figure 4). For instance, findings in simulation studies support the negative impact of inflammatory cells at the implantation site,^191^ as they deplete glucose. This causes misreading of the actual glucose levels in blood, limiting the overall device performance.

Orchestrating the host tissue response, macrophages are pivotal at controlling the device function. A comparative study implanting CGM sensors in wild‐type and macrophage‐depleted mouse models proved that following 4 weeks of implantation, the former model caused accumulation of macrophages that limits the sensor functionality. In contrast, the latter enhanced the device performance, indicating that sensor impairment could be macrophage‐associated.^192^ In another study, the same group reported that, after peritoneal injections of mouse macrophages in the proximity of CGM in mouse model, glucose levels measured by the sensor were lower than blood glucose levels.^193^ CCL2 and CCR2 are leukocyte chemotactic factors that contribute to monocyte/macrophage activation and eventually the formation of FBGCs. Utilizing CCL2 and CCR2 knockout mouse models, the same group reported that the monocyte/macrophage accumulation was significantly reduced compared to the wild‐type mice.^194^ Furthermore, the relative low difference between sensor glucose level readout and blood glucose levels indicates that sensor accuracy was improved. This might also be attributed to the indirect inhibition of TGF‐β signaling receptor that contributes to the reduced analyte diffusion in sensors.^195^

Alternatively, zwitterionic polymer modification on commercial CGMs abrogated the cross‐talk between inflammatory cells and sensor electrode surface, resulting in a reduced capsule formation compared to the uncoated control implant.^123^ The treatment reduced the immune response towards the sensor in mice and NHP. In addition, no recalibration was needed, and the sensors accurately measured glucose levels throughout the study.

To summarize, drug and analyte diffusion within the surrounding of the device can be significantly impaired in the event of severe and uncontrolled FBR to the device or to the drug formulation, especially in the long‐term. While it is intuitive that extremely collagen dense and poorly vascularized fibrotic tissue can be a physical obstacle to molecules diffusion and biodistribution, it appears that a milder FBR can still grant satisfactory device performances. However, more research is needed to study how different characteristics of the fibrotic capsule affect molecules diffusion.

### 3.2 The effect of fibrosis on cell encapsulation devices

Cell transplantation is a promising approach that entails administering living cells to patients as replacement therapy to treat various disorders. Transplanted cells can deliver therapeutic molecules in a sustained fashion or in response to stimuli. Typically, these cells can originate either from a donor or can be engineered or obtained from animal sources. Consequently, in various cases, cell transplantation requires some extent of immune protection to prevent graft rejection. To this end, cell encapsulation devices emerged as platforms to house transplanted cells in an optimal microenvironment. These devices must be carefully designed to create an immune‐privileged milieu while still allowing for a facile exchange of nutrients, analytes, and therapeutic molecules to and from the cells. The FBR heightens challenges in balancing these aspects towards the device. Unmodulated FBR can lead to a dense fibrotic capsule and consequently limiting oxygen and nutrient supply. This can ultimately affect graft viability and function.^63^, ^183^

Most allogeneic cell encapsulation devices rely on the principle of immune isolation to prevent graft rejection. This can be achieved by encasing the cells in polymeric capsules or semipermeable membranes. Thus, immune cells are physically hindered from attacking the transplanted cells, while allowing diffusion of oxygen, nutrients, analytes, and therapeutic molecules in the device. Other approaches allow for direct vascularization of the graft, albeit exposing it to immune cell interaction. These platforms use systemic or local administration of immunosuppressive drugs to avoid the destruction of the graft by the immune system.^22^, ^196^, ^197^, ^198^, ^199^

The first crucial step in designing a cell encapsulation system is selecting an optimal material.^200^ Alginate is the prevalent choice for cell microencapsulation platforms, however, adjustments are required to achieve solid engraftment and prevent fibrotic isolation.^63^, ^183^, ^201^ For example, chemical modification of alginate with triazole‐thiomorpholine dioxide (TMTD) elicited a weaker FBR in immunecompetent mice.^202^ Encapsulation of human embryonic stem cell‐derived β cells in TMTD alginate microcapsules allowed for long‐term glycemic control in diabetic mice. Moreover, pancreatic islets encapsulated with chitosan‐modified alginate capsules showed reduced pericapsular fibrosis and promoted type‐1 diabetes (T1D) reversal for up to a year in immunecompetent dogs.^203^ In another study, zwitterionically modified alginate mitigated cellular overgrowth and fibrosis across different species.^204^ Encapsulating islets in the modified alginate improved glycemic control in mice. Furthermore, the incorporation of immunomodulatory molecules in alginate capsules demonstrated to be for long‐term engraftment and function of insulin‐producing cells.^205^, ^206^ In particular, Farah et al. formulated an alginate hydrogel combined with crystalline GW2580, a colony‐stimulating factor‐1 receptor (CSF‐1R) inhibitor, and evaluated its effect for xenogeneic islet transplantation.^207^ Alginate‐GW2580 microcapsules achieved long‐term release of the immunomodulator which reduced fibrosis and facilitated glycemic control in mice when used for islets transplantation in mice. As previously discussed, the size of alginate capsules can influence the FBR and therefore affect the outcome of T1D treatments that employ encapsulated islets. For example, bigger capsules (1.5 mm in diameter), which elicited a mild FBR, promoted and maintained normoglycemia for up to 140 days in diabetic mice.^146^ Contrarily, smaller capsules (0.5 mm in diameter) were not as effective in reverting hyperglycemia. In fact, high cellular deposition and fibrosis observed on the capsule surface appear to have affected islets viability and function.

Several other materials and strategies have been explored to minimize FBR on encapsulated cells. For instance, hyaluronic acid (HA) based hydrogels can be a valid alternative to alginate.^208^ HA is a component of the ECM used in combination with collagen or zwitterionic compounds to prevent biofouling and improve cytocompatibility.^209^ Microspheres made of HA and denatured collagen derived hydrogel were used to encapsulate pancreatic islets.^208^ The encapsulated cells, transplanted in the omentum of diabetic rats, could maintain euglycemia for up to 52 weeks with minimal fibrosis. A HA hydrogel employed in a cell macroencapsulation system in combination with VEGF‐containing microspheres, increased the stability and maturity of the surrounding capillaries without aggravating the FBR.^210^ PEG grafting on pancreatic islets as a different strategy can reduce immunogenicity and, therefore, enhance the therapeutic efficacy of the graft.^206^, ^211^, ^212^

Macroencapsulations employ semipermeable polymeric membranes to encapsulate a large number of cells in one device,^183^, ^213^ which supports graft retrievability. However, a gradient of oxygen and nutrients can form within the device and impair cell viability and function. Therefore, tuning FBR is fundamental to balance fibrosis reduction around the implant while still obtaining angiogenesis in its proximity.^214^ Weaver et al. enveloped an alginate macroencapsulation device with a vasculogenic biodegradable hydrogel to enhance vascularization in the vicinity of the device.^215^ This implementation resulted in improved islet viability in a diabetic rat model. Similarly, MSCs can be coencapsulated or cotransplanted with the therapeutic cells to exploit their immunomodulatory and angiogenic properties.^22^, ^198^, ^216^, ^217^ In addition, anti‐inflammatory macrophages and dendritic cells secrete essential factors for β‐cell survival, proliferation, and insulin release. Therefore, preventing immune system infiltration entirely can be detrimental for the engrafted cells.^218^, ^219^ Moreover, semipermeable membranes may allow for shed antigens to diffuse outside the implant and cause an immune response against the implant.^220^ Likewise, cytotoxic molecules can cross the membrane and induce immune toxicity in the graft.^221^ These effects presumably contributed to the FBR‐induced hypoxia that led the insulin‐producing cells macroencapsulation device developed by ViaCyte to failure in a phase I clinical trial.^222^

A different scenario needs to be considered for cell encapsulation technologies in the context of cancer immunotherapy. In this application, the devices are usually implanted for less than a month. Therefore, the long‐term viability of the cells is not a concern. Contrarily to other cell encapsulation systems, these platforms aim to boost a strong inflammatory reaction resulting in a milieu conducive for antitumor immune response.^223^

Based on the specific application or approach, the FBR can be exploited in different manners. Generally, macroencapsulation devices require a certain degree of inflammatory response to generate neovasculature that can deliver oxygen and nutrient to the graft. Vice versa, microencapsulation platforms aim at evading the immune response entirely to prevent the formation of a fibrotic capsule that will limit oxygen and nutrient diffusion to the cells.

## 4 NOVEL BIOMIMETIC STRATEGIES TO MODULATE FBR

Conventional strategies for mitigating FBR and scar tissue formation employ immunosuppressive agents such as DEX.^224^ However, occurrences of detrimental side effects of those agents are inevitable. Instead, nature‐inspired biomimetic surface modifications are attractive, widely investigated options to stealth the implants from the immune system and promote tissue‐device integration. In this section, we will cover the recent biomimetic approaches for implantable biomaterials (Table 2).

**TABLE 2:** Summary of biomimetic strategies for FBR modulation

Biomimetic Strategy	Material	Implant type	Methods	Surface modification effects	References
Zwitterionic	PCMA hydrogel	Hydrogel for multiple applications	In vivo testing in mice for >3 months	Avoids macrophage recognition and FC formation	124
	Phosphoserine hydrogel discs	Hydrogel for drug delivery	In vitro testing in culture with fibrinogen	Antiadhesive properties towards proteins	225
	Triazole‐modified alginate	Microspheres for islets encapsulation	In vivo testing in mice and NHP for 4 weeks	Low immune cell recruitment and prevented macrophage activation	200
	Sulfobetaine modified alginate	Microspheres for islets encapsulation	In vivo testing in mice for 200 days, pigs and dogs for 90 days	Mitigate cellular overgrowth and fibrous tissue around transplanted islets	204
	Triazole hydrogel	Hydrogel for islets encapsulation	In vivo testing in mice for 4 weeks	Anti‐biofouling properties and improved mechanical stability	226
	Polysulfobetaine and poly(carboxybetaine) hydrogels	Tissue scaffolds	In vitro incubation with serum proteins and in vivo testing in mice for 1 year	Antifouling properties and resistance against fibrosis	227
	2‐Methacryloyloxyethyl phosphorylcholine (MPC)	Coating on CGM devices	In vivo testing in mice and NHP for up to 8 days	Reduction of proinflammatory markers on implantable glucose sensor and mitigated signal‐to‐noise ratio	123
		Silicone breast implant coating	In vitro incubation in BSA and in vivo implantation in pigs for 6 months	Reduction of BSA adsorption, reduction of proinflammatory factors and inhibition of fibrous tissue formation	228
ECM molecule coating	Gelatin–hyaluronic acid	PDMS surface coating	In vivo testing in rats for 2 months	Attenuated fibrotic tissue formation	229
	HA and heparin	Surface modification for silicon wafers	In vitro culture with THP‐1‐derived macrophages	Supression of the NF‐κB signaling pathway	230
Protein coating	Gelatin	Electrospun membrane coating for implantable glucose sensors	In vivo testing in rats for 3 weeks	Reduced fibrosis and improved sensor sensitivity	231
	Fibronectin and IL‐4	Functionalization and coating of hollow PES fibers for cell encapsulation	In vivo testing in mice for 14 days	Reduced fibrotic capsule thickness in the early stage of inflammation and improved angiogenesis and encapsulated cells survival	232
	Pectin	3D‐printed alginate‐pectin construct for cell encapsulation	In vivo testing in mice for 4 weeks	Decreased fibrotic capsule thickness and reduced cellular infiltration at the implantation site Slight improvement in islet xenograft survival	233
	Silk fibroin and mechano growth factor	Decoration of an electrospun PCL scaffold	In vivo testing in rats for 4 weeks	Thinner fibrotic tissue formation and improved islet encapsulation in a microcapsule device by reducing immune cell infiltration and triggering an anti‐inflammatory pathway	234
	Colony‐stimulating factor‐1 (CSF‐1)	Surface functionalization of PLLA scaffolds	In vivo testing in mice for 4 weeks	Reduced proinflammatory cytokine and increased wound‐healing macrophages	235
Surface patterning	Patterned PDMS mimicking breast tissue	Breast implant surface modification	In vitro culture with THP‐1 macrophages	Enhance M2 polarization and reduced TNF‐α levels	236
Implant wrapping	Biocellulose	CIED wrap	In vivo testing in minipigs for 12 months	Reduced fibrotic tissue formation	237
Scaffold modification	Melatonin, thiolated HA and collagen I	PCL/melatonin fibers + thiolated HA/collagen I scaffold for muscle regeneration	In vivo testing in rats for 8 weeks	Promoted cell proliferation on the scaffold and enhanced M2 polarization leading to muscle regeneration	238
	Endometrial MSCs	Poly‐l‐lactic acid‐co‐poly ε‐caprolactone nanofibrous transvaginal mesh loaded with endometrial MSCs	In vivo testing in mice for 6 weeks	Enhanced angiogenesis, collagen production, and M2 polarization	239
Surface modification	Human cardiomyocytes	3D nonporous carbon fiber electrodes embedded in human cardiomyocytes	In vitro testing with tissue engineered spontaneously beating human cardiac patches	Reduced FBR and have the regenerative capacity in vitro	240

Abbreviations: 3D, three‐dimensional; BSA, bovine serum albumin; CGM, continuous glucose monitoring; CIED, cardiovascular implantable electronic device; FBR, foreign body response; IL‐4, interleukin‐4; MSC, mesenchymal stem cell; NF‐κB, nuclear factor kappa B; NHP, nonhuman primate; PCL, polycaprolactone; PCMA, poly(carboxybetaine methacrylate); PDMS, polydimethylsiloxane; PES, polyethersulfone; PLLA, poly‐l‐lactic acid; TNF‐α, tumor necrosis factor‐α.

### 4.1 Zwitterionic molecule coating

Zwitterionic polymers that alleviate FBR have become attractive candidates as a coating strategy for implantable devices.^241^ For instance, zwitterionic poly(carboxybetaine methacrylate) hydrogels can be effective for more than 3 months to avoid macrophage recognition and fibrotic capsule formation in mice.^124^ Similarly, inspired by the naturally occurring immunological tolerance mediated by phosphoserine (PS), zwitterionic PS hydrogel discs demonstrate antiadhesive properties when cultured in fibrinogen‐rich culture in vitro.^225^

Triazole‐zwitterionic (TR‐ZW) hydrogels used for islet encapsulation and transplantation can also reduce FBR.^242^ In addition, they can trigger new blood vessel formation for oxygen and nutrient exchange while preserving the normoglycemic conditions in diabetic mice. This indicates that triazole–zwitterionic hydrogels can support graft function. In another study, Vegas et al. created an extensive combinatorial alginate hydrogel library to evaluate the chemically modified materials that reduced FBR in NHP. Alginates with modified triazole derivatives exhibited low immune cell recruitment at the material surface and prevented macrophage activation.^200^ Similarly, rat islet encapsulated in sulfobetaine modified alginate microcapsules can mitigate cellular overgrowth and fibrous tissue in pigs, mice, and dogs.^204^

These studies suggest that zwitterionic functional surface coatings may hold promise on immunomodulation. However, due to the high hydrophilicity of the zwitterionic‐based implants, such systems bear a mechanically weak profile, affecting long‐term resistance against FBR and capsular structure around the implant.^227^ In this regard, Liu et al. developed a TR‐ZW hydrogel that exhibited improved mechanical robustness while maintaining similar anti‐biofouling properties compared to conventional zwitterionic hydrogels. Rat islets encapsulated in TR‐ZW hydrogel improved diabetes correction in mice.^226^ Similarly, a zwitterionic elastomeric network that consists of polysulfobetaine (PSB) and poly(carboxybetaine) (PCB) hydrogels demonstrated antifouling feature after incubation with serum proteins. Moreover, after subcutaneous implantation in mice for a year, PSB/PCB hydrogel network retained its resistance against fibrosis.^227^

2‐Methacryloyloxyethyl phosphorylcholine (MPC) is a water‐soluble compound that possesses phosphorylcholine hydrophilic groups present in biological membranes.^243^ Due to antifouling and low inflammatory anticell adhesive features,^121^, ^244^, ^245^ MPC grafting onto the PDMS implants demonstrates an inhibitory effect in fibrous tissue formation in a rat model. Utilizing heat‐induced polymerization as a more effective grafting method, a remarkable reduction in BSA adsorption (by 55%) compared to the virgin PDMS in vitro was observed.^228^ When implanted in the submuscular space in a pig model, MPC decorated silicone breast implants can significantly reduce proinflammatory associated α‐SMA and TGF‐β levels as well as fibrous tissue formation for 6 months.^228^ Similarly, when applied on Medtronic subcutaneous CGM device surfaces, poly‐MPC demonstrated a significantly reduced level of proinflammatory markers that may be associated with sensing noise. Overall, MPC shows a promising approach to mitigate the signal‐to‐noise ratio and improve device performances when implanted in mice, healthy and diabetic NHP.^123^

However, during the surface coating process, polymerization time and monomer concentration are challenging requirements for biocompatibility. For instance, despite increased surface hydrophilicity with plasma treatment, longer reaction time and monomer concentration above specific concentration clogged the mesh pores during the modification of polypropylene surgical meshes with PMC polymer.^246^

Overall, finely optimizing the density of the polymer coating on the biomaterial and grafting zwitterionic polymers on the implantable devices suggests a great potential to hamper the FBR, which may pave the way for the clinical translation of such engineered surfaces.

### 4.2 Protein/ECM molecule coating

Inspired by the ECM structure, it is possible to generate implantable surfaces that hamper cellular recognition and cell activation, hindering fibrotic capsule formation around the implants. Coating the implant surface with ECM molecules that endorse the integration of the implants with the host tissue boosts biocompatibility by dampening the proinflammatory signaling cascades.^229^ Tan et al. reported that polyethersulfone (PES) encapsulation membranes coated with ECM protein, fibronectin (FN) and IL‐4, generate a thinner fibrotic capsule (6.4 ± 2.24 μm) compared to the only FN coated and control PES implants (13.5 ± 4.11 and 14.7 ± 2.74 μm, respectively). This effect, observed after 14 days of subcutaneous implantation in mice, may be explained by the M2 polarizing effect of IL‐4. Finally, coated implants improved islet cell engraftment to the membranes and angiogenesis.^232^

Negatively charged glycosaminoglycans (GAGs) are one of the critical components of ECM. Covalent attachment of the GAGs onto the implantable devices showed enhanced anti‐inflammatory effect due to regulatory T cell activation.^247^ In an in vitro study, researchers assembled hyaluronic acid and heparin, from the GAG class, on amino‐terminated silicone and glass substrates leading to the downregulation of nuclear factor kappa B subunit p65, a protein associated with macrophage activation.^230^ In another study, gelatin‐coated PU‐based electrospun fiber membranes with a diameter of 1.54 μm showed a reduced fibrous structure for 3 weeks after subcutaneous implantation in a rat model.^231^ However, the instability of gelatin‐coating for longer times can be challenging in applications where sensor sensitivity is relevant. Modifying the PDMS surface with gelatin combined with hyaluronic acid (HA) can provide mechanical stability, adjustable degradation property, and hydrophilicity similar to soft tissue while attenuating the fibrotic tissue formation in a rat model for 2 months.^229^

Blood‐contacting devices such as cardiovascular stents have limited long‐term clinical success due to the in‐stent restenosis, a series of events including thrombosis, platelet aggregation on the metal stent, and reduced re‐endothelialization.^248^ Heparin, the most clinically used anticoagulant molecule, has been known to prevent the early stage of thrombosis.^249^ Utilizing artificial ePTFE vascular grafts coated with poly(l‐lactide‐co‐ε‐caprolactone) (PLCL), a biodegradable and biocompatible elastomeric polymer incorporated heparin/substance, enhanced angiogenesis and recruitment of MSCs, smooth muscle cells, M2 polarization after 4 weeks of implantation in rat models.^250^ Another strategy is the biofunctionalization of PTFE vascular grafts with CD47, SDF‐1α, and heparin via plasma immersion ion implantation (PIII). Roughening the surface via PIII facilitates the attachment of molecules. Unlike bare PTFE grafts, functionalized grafts have shown an anti‐inflammatory surface that is not favorable for macrophage adhesion in vitro. Furthermore, sustained release of the chemokine SDF‐1α that helps recruit circulating endothelial progenitor cells, conferring long‐term patency to the graft.^251^ Similarly, a synergistic effect of nitric oxide and CD47 peptide immobilized on the surface of PU‐coated silicone tubing showed a reduced thrombosis and early‐stage inflammatory response both in in vitro and ex vivo models.^252^ CD47 on 316L grade stainless steel stents demonstrated a reduction in early‐stage platelet formation and macrophage activation, and 30% reduced restenosis in the rat carotid artery model.^253^

Pectin, a natural polysaccharide, blocks toll‐like receptor (TLR) signaling,^254^ another critical stage for macrophage recruitment to the implantation site.^255^ Incorporating pectin with a low degree of methyl esterification on cross‐linked alginate cell‐laden hydrogel, Hu et al. showed a significant decrease in the fibroblast thickness and elimination of the immune cell filtration after 28 days of subcutaneous implantation in mice.^233^ The same group found that a low degree of methylated pectin incorporated in alginates can effectively prevented TLR‐2 activation through electrostatic interaction and consequently suppressed immune activation. The material, used to encapsulate rat islets, prolonged their survival in a xenogeneic graft in mice compared to alginate and high degree pectin capsules.^256^ However, it is still unclear how the degradation of the hydrogel compound will affect the long‐term cell survival in the cellular envelope and suppression of overt fibrosis.

Due to its high biocompatibility,^257^ mechanistic and porous features, silk protein has been broadly used as a biomaterial to construct drug delivery matrixes and tissue engineering scaffolds.^258^ For example, in a recent study, a group designed a pancreatic islet encapsulating microcapsule with a shell made of alginate or agarose gel, and a core that was incorporated with a silk scaffold encapsulating the islet cell. This creates a more realistic ECM‐like structure that is crucial for long‐term islet survival. Furthermore, creating an additional interior layer with silk can prevent immune cell filtration and induced an anti‐inflammatory pathway.^259^ The bilayered RGD peptide are silk tissue‐engineered vascular grafts that mimic the blood vessel structure.^258^ An inner porous layer mirrors the tunica media while the outer electrospun layer is similar to the adventitia. The implant promoted graft patency with a minimal fibrous tissue formation in Lewis rats 8‐week postimplantation. In another study, Song et al. functionalized the LBL silk fibroin (SF) modified PCL nanofibrous scaffolds with mechano growth factor‐1 (MGF‐1), an alternative splicing product of insulin growth factor‐1. Through the upregulation of the anti‐inflammatory signaling cascade such as signal transducer and activator of transcription 6 activation, MGF‐1/SF/PCL scaffolds induce thinner fibrotic tissue formation and higher M2/M1 ratio than the SF/PCL and bare PCL scaffold post‐28‐day implantation in rats.^234^

Poly‐l‐lactic acid (PLLA), with a low biodegradation rate and mild immune response, has been clinically used as an implantable material. However, increasing the immunomodulatory effect is crucial to increase the long‐term fate of the PLLA‐based devices. Immobilizing the macrophage CSF‐1, a hematopoietic growth factor responsible for tissue repair on the PLLA scaffolds, can enhance biocompatibility with reduced IL‐1β, TNF proinflammatory cytokine levels, and increased CD68^+^ and CD206^+^ levels up to 28‐day postimplantation in IL‐1β reporter C57BL/6 mice.^235^

Taken together, these findings confirm the short‐term effectiveness of bioactive molecule coating in directing the immune cascade pathway towards the tissue repair phase. However, long‐term fate of the coating remains challenging and requires extensive design testing both in vitro and in vivo.

### 4.3 Other biomimetic approaches

Patterning the implant surface can influence protein adsorption and cell adhesion. Surfaces that mimic the natural tissue texture can significantly reduce the curvature at the biomaterial–tissue interface, leading to reduced fibrosis and cellular morphology.^240^ Using a 3D grayscale photolithography approach, Bar et al. were the first to show that patterned PDMS implants that mimic natural breast tissue surface can enhance the M2 polarization while reducing M1‐associated TNF‐α levels in vitro THP‐1 monocyte cell line culture.^236^ Furthermore, microgrooves (~24 μm) and nanofibrillar structures (~700 nm) that mimic vascular smooth muscle cell morphology on the 316L vascular stents via femtosecond laser ablation method can enhance re‐endothelization and reduce in‐stent restenosis for 90 days in a rabbit model.^260^ An interesting approach to enhance the long‐term performance of cardiovascular implantable electronic device implants is coating surfaces with biocellulose (BC) membrane as conformal wrapping protection around the devices. Modification of the implant surfaces with nonbioresorbable BC significantly reduced the fibrotic tissue formation (66% reduction in tissue thickness compared to the unmodified devices) in clinically relevant minipig model after 12 months.^237^

Incorporating melatonin (MLT)‐loaded PCL electrospun fibers into thiolated hyaluronic acid/collagen hydrogels is another strategy to generate a biomimetic scaffold. To mimic the native muscle fibers, highly ordered electrospun fibers were generated with a diameter range between 400 and 700 nm. The scaffold was suitable for muscle cell proliferation in a volumetric muscle loss rat model on the tibialis anterior muscle regeneration. After 8 weeks of implantation, PCL/MLT showed an enhanced M2 polarization, suggesting that MLT has an anti‐inflammatory effect.^238^

Another strategy that mimics the natural ECM is the incorporation of MSCs into the implant. MSCs are clonogenic, multipotent cells that can differentiate into various cells. This potential makes them attractive for use in regenerative medicine.^261^, ^262^ Bioengineering, PLCL nanofibrous mesh (P nanomesh) with a diameter of 585 nm closely resembles the human vaginal microstructure at the nanoscale.^239^ This mesh, comprised of collagen fibril structures with endometrial MSCs, showed enhanced angiogenesis and collagen production, indicators of tissue integration of the meshes even after 6 weeks of subcutaneous implantation in mice compared to P nanomesh alone. The synergistic effect of surface texture and MSC activation promotes upregulation of CD206 expression, showing M2 macrophages polarization.

Moreover, biomimetic strategies can aid in overcoming FBR barrier faced by titanium nitride pacemaker electrodes. 3D hybrid nonporous carbon fiber electrodes for example can induce M2 macrophage proliferation and reduce FBR with a regenerative capacity in vitro compared to the 2D smooth TiN layers.^240^

Notably, these findings suggest that mimicking ECM in designing biocompatible implantable devices is a valid strategy to overcome intense FBR. However, the long‐term stability of coating materials is still unclear, and requires extensive research.

## 5 CONCLUSION AND FUTURE CONSIDERATIONS

Implantable devices have been clinically employed for decades. However, there is no gold standard to prevent or modulate the FBR. Therefore, understanding the mechanisms of fibrotic tissue response to implantable devices is fundamental. Implant parameters, including surface wettability, topography, shape, and size, determine the degree of protein adsorption, and the proinflammatory response which may ultimately result in scar tissue formation. Considering the potential outcomes from a clinical perspective, designing innovative implantable materials to control the protein adsorption process and avoid the immune response is crucial to elucidate the implant's performance for a prolonged period. However, the implant properties that affect FBR are tightly interconnected. Multiple studies reveal that an increase in surface roughness can generate air pockets within the grooves of the surface and lead to higher hydrophobicity. Conversely, the liquid can penetrate the grooves at lower roughness, producing more hydrophilic surfaces.^263^, ^264^, ^265^ Furthermore, the wettability of a material is highly dependent on its surface chemistry. The charged or polar functional groups exposed on the surface, either naturally, or due to a superficial treatment, determine the overall charge that interacts with water molecules.^266^, ^267^ In addition, functionalization or optimization in formulation aimed at improving the biocompatibility of a material can significantly alter its mechanical properties, thus affecting the overall stiffness and durability of the implant.^140^, ^268^ Therefore, different biomaterial features should be rigorously characterized to achieve the desired FBR mitigation and preserve device functionality.

The International Standard ISO 10993‐1 principles for the biological evaluation of medical devices provide essential guidelines for in vitro/in vivo testing.^269^ In vitro biocompatibility studies are mainly performed in 2D cell culture, failing to mimic the complex 3D physiological environment. Macrophages in 2D cell culture models show different phenotypes and responses to stimuli compared to in vivo settings.^270^ In addition, short‐term biocompatibility assessment (less than a month of evaluation) may lead to biased outcomes.^271^ Therefore, biomaterial compatibility test duration should be carefully selected and in vitro findings should be validated in vivo.^272^ To this extent, selecting the most suitable animal model to mimic the FBR in humans is paramount, as different species or strains can produce substantially distinct FBR. Eventually, scaling the platform to clinical translation requires further consideration. Variation in immune response among individuals, which can be related to underlying conditions or aging, needs to be accounted for. Creating a dynamic immune cell model in a lab on a chip platform can be a solution for a personalized evaluation of FBR which will be a promising strategy to reduce laboratory animal use.^273^ Finally, recent progress in implementing biomimetic strategies to control FBR holds promises towards curtailing the immune response to implantable devices. For instance, CorNeat Vision's biomimetic nonbiodegradable implant that mimics ECM topography is currently under clinical trial (NCT04485858). However, much progress is required, particularly in implementing high throughput screening platforms in the early stage of device development to pave the way for clinical translation.

## AUTHOR CONTRIBUTIONS

**Simone Capuani:** Conceptualization (equal); visualization (equal); writing – original draft (lead); writing – review and editing (equal). **Gulsah Malgir:** Conceptualization (equal); visualization (equal); writing – original draft (lead); writing – review and editing (equal). **Corrine Ying Xuan Chua:** Conceptualization (equal); supervision (equal); writing – review and editing (equal). **Alessandro Grattoni:** Conceptualization (equal); funding acquisition (lead); supervision (lead); visualization (equal); writing – review and editing (equal).

## CONFLICT OF INTERESTS

Simone Capuani, Corrine Ying Xuan Chua, and Alessandro Grattoni are inventors of intellectual property licensed by NanoGland LLC. Gulsah Malgir declares no conflict of interest.

### PEER REVIEW

The peer review history for this article is available at https://publons.com/publon/10.1002/btm2.10300.

## Data Availability

Data sharing not applicable to this article as no datasets were generated or analyzed during the current study.
